# Antifungal Activity and Synergism with Azoles of Polish Propolis

**DOI:** 10.3390/pathogens7020056

**Published:** 2018-06-19

**Authors:** Katarzyna Gucwa, Barbara Kusznierewicz, Sławomir Milewski, Patrick Van Dijck, Piotr Szweda

**Affiliations:** 1 Department of Pharmaceutical Technology and Biochemistry, Faculty of Chemistry, Gdańsk University of Technology, Narutowicza 11/12 Str., 80-233 Gdańsk, Poland; katarzyna.gucwa@pg.edu.pl (K.G.); slawomir.milewski@pg.edu.pl (S.M.); 2 Department of Chemistry, Technology and Biotechnology of Food, Faculty of Chemistry, Gdańsk University of Technology, Narutowicza 11/12 Str., 80-233 Gdańsk, Poland; barbara.kusznierewicz@pg.edu.pl; 3 VIB-KU Leuven Center for Microbiology, Kasteelpark Arenberg 31 bus 2438, 3001 Leuven, Belgium; patrick.vandijck@kuleuven.vib.be; 4 Laboratory of Molecular Cell Biology, KU Leuven, Kasteelpark Arenberg 31, bus 2438, 3001 Leuven, Belgium

**Keywords:** propolis, *Candida* spp., biofilm, resistance, synergism

## Abstract

The aim of our work was to check if one of the products of natural origin, namely honey bee propolis, may be an alternative or supplement to currently used antifungal agents. The activity of 50 ethanolic extracts of propolis (EEPs), harvested in Polish apiaries, was tested on a group of 69 clinical isolates of *C. albicans*. Most of the EEPs showed satisfactory activity, with minimum fungicidal concentrations (MFC) mainly in the range of 0.08–1.25% (*v*/*v*). Eradication of biofilm from polystyrene microtitration plates in 50% (MBEC_50_, Minimum Biofilm Eradication Concentration) required concentrations in the range of 0.04% (*v*/*v*) to more than 1.25% (*v*/*v*). High activity was also observed in eradication of biofilm formed by *C. glabrata* and *C. krusei* on the surfaces of PVC (Polyvinyl Chloride) and silicone catheters. EEPs at subinhibitory concentrations inhibited yeast-to-mycelia morphological transformation of *C. albicans* in liquid medium and mycelial growth on solid medium. A synergistic effect was observed for the action of EEP in combination with fluconazole (FLU) and voriconazole (VOR) against *C. albicans*. In the presence of EEP at concentrations as low as 0.02%, the MICs of FLU and VOR were 256 to 32 times lower in comparison to those of the drug alone. Evidence for the fungal cell membrane as the most probable target of EEPs are presented.

## 1. Introduction

An increasing number of fungal infections is an alarming problem, especially for immunocompromised patients. *Candida albicans* species have been recorded as the most frequent cause of life threatening disseminated candidiasis, representing up to 60% of isolates. However, recently, non-*albicans Candida* spp. resistant to conventional treatments has emerged as prevalent causes of candidiasis, including *C. parapsilosis*, *C. tropicalis* and *C. glabrata* [1,2]. Moreover, *C. albicans*, other *Candida* spp. as well as other pathogenic yeasts have developed different mechanisms of resistance against antifungal agents, including mutations of genes coding for molecular targets of azoles, echinocandins or flucytosine and overexpression of drug transporters [3,4,5]. Many nosocomial-acquired infections are associated with the use of medical devices (e.g., catheters), which are the sites for fungal biofilm development. Cells living in biofilm produce an extracellular matrix (ECM) composed of a combination of macromolecules, including polysaccharides, proteins, nucleic acids, and lipids. The ECM of fungal biofilm has been shown to provide numerous functions, including cellular cohesion, community structure, nutritional resource, and, especially important from the clinical point of view, protection from xenobiotics, antimicrobials, and the host immune system [6]. As a consequence, both systematic and biofilm related types of candidiasis are difficult to cure and require prolonged treatment, which often leads to development of resistance of yeast to common antifungal drugs. All presented aspects above clearly indicate that there is an urgent need to develop new antifungal agents, effective, inexpensive and not covered by currently existing mechanisms of resistance. Natural products of different origins constitute a promising but still underestimated group of potential antifungal agents—among them, honey bee propolis seems to be especially interesting. Propolis is a highly sticky, resinous substance of complex chemical composition. Some of its ingredients, mainly polyphenols and flavonoids, exhibit high antimicrobial activity. As a consequence, bees use this product as a hive disinfectant [7]. The exact composition of raw propolis depends on the source of its harvest. In general, it consists of 50% resin, 30% vegetable and bee wax, 10% essential oils, 5% pollen and 5% various other substances, including organic pollutants [8]. Propolis-containing extracts are supposed to have antiseptic, antifungal, bacteriostatic, astringent, diastolic, anti-inflammatory, anesthetic, and antioxidant properties [8,9,10]. In this work, we have tested the activity of ethanolic extracts of propolis (EEPs) gathered in northern Poland towards planktonic and biofilm related cells of *Candida* spp. Additionally, we have investigated their synergistic interactions with antifungal drugs and a possible mode of antifungal action.

## 2. Results

### 2.1. Anti Candidal In Vitro Activity of Propolis Ethanolic Extracts

Ethanolic extracts prepared from 50 propolis samples (EEPs) collected from different regions of northern Poland were tested for their anti *Candidal in vitro* activity against planktonic *C. albicans* (five strains: one reference strain and four clinical isolates) and *C. glabrata* (reference strain) cells. In some cases, the MIC values could not be unequivocally determined because of the presence of sediments occasionally present in wells containing EEPs. Therefore, the minimum fungicidal concentrations (MFCs) values were determined. Results of this assay are presented in Table 1. For *C. albicans* strain ATCC 10231 seven samples of EEPs had an MFC value above 2.5% (*v*/*v*), eight had an MFC value equal to 2.5%, for 12 samples, the MFC was 1.25%. The MFC value equal to 0.63% was established for 15 EEPs and 0.31% for eight EEPs. Taking into consideration all the reference strains, the MFC equal or higher than 2.5% was obtained in 112 cases. The most frequent MFC value was 0.63% (103 cases), and the value 0.31% was seen in 25 cases (Table 1).

The results of this test were the basis for the selection of extracts for the next stages of research. Unfortunately, in the case of some EEPs, recognized as most active, we disposed with samples of limited volume. After running out of the whole sample, we replaced it with another EEP that exhibited comparable activity. In general, only the products with MFC values equal to 0.31 or 0.63 were used in the following research.

**Figure 1:**
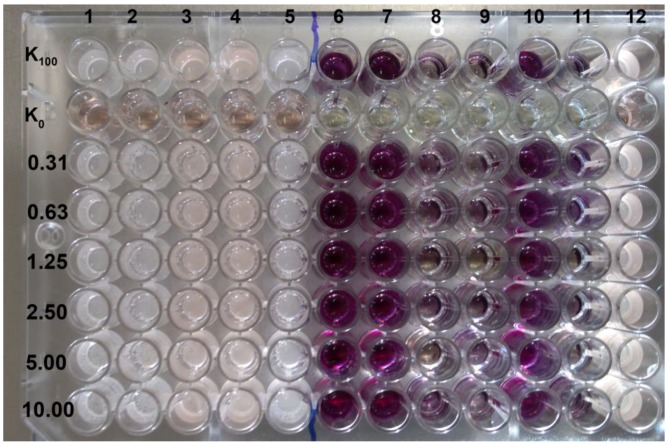
Effect of ethanol (solvent used for propolis extraction) on MIC values and biofilm eradication. Ethanol was tested in the range of concentration 10–0.31% (*v*/*v*) on two reference strains *C. albicans* ATCC 10231 (columns with numbers 1, 2, 5—MIC evaluation, 6, 7, 10—MBEC_50_ evaluation with MTT (3-(4,5-dimethylthiazol-2-yl)-2,5-diphenyltetrazolium bromide) assay) and *C. glabrata* DSM 11226 (numbers 3, 4, 12 and 8, 9, and 11, respectively).

Six out of 50 EEPs tested, assigned as 17, 21, 27, 15 b, 18 b, and 23, exhibiting the highest antifungal activity (MFC in the range 0.31–0.63% (*v*/*v*) against *C. albicans* ATCC 10231), were chosen for further evaluation of their potential in terms of MFC values against 69 *C. albicans* clinical isolates. The data obtained are presented in Table 2. For 77% of isolates, MFC values were 0.31 or 0.63% (*v*/*v*). Only in three cases was the MFC value ≥ 2.5% (*v*/*v*) observed (Table 2). Controls of solvent used for propolis extraction showed that concentration of ethanol up to 10% (*v*/*v*) does not affect the growth of *C. albicans* ATCC 10231 and *C. glabrata* DSM 11226 (Figure 1).

### 2.2. Kinetics of the Fungicidal Action of EEPs

**Figure 2:**
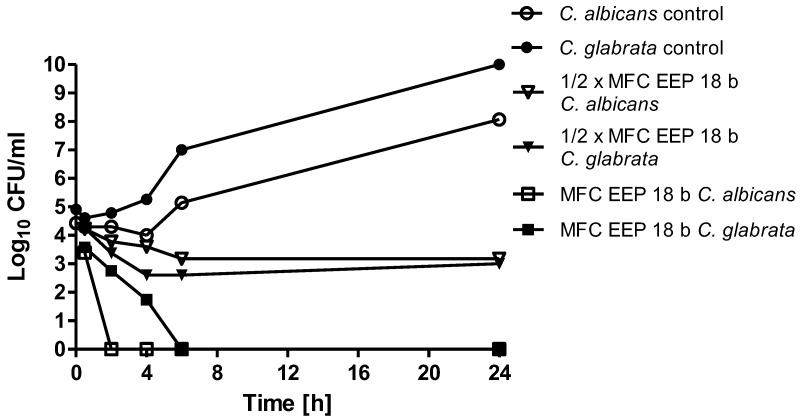
Time kill analysis. *C. albicans* ATCC 10231 and *C. glabrata* DSM 11226 cells were first refreshed on YPD (Yeast extract Peptone Dextrose) agar plates to stationary phase. OD_660_ = 0.1 was established, suspension was diluted 10 times and grown in RPMI (Roswell Park Memorial Institute)-1640 medium in the presence of EEP 18 b at concentrations 0.31% (*v*/*v*) (½× MFC, usually equal to MIC) and 0.63% (*v*/*v*) (MFC)—equal for both species. EEPs were added at time zero.

The time kill curves for the most active EEPs acting on *C. albicans* or *C. glabrata* cells at concentrations corresponding to ½× MFC or MFC values are shown in Figure 2.

At concentrations corresponding to ½× MFCs, the 2–3 log10-fold reduction in the cell count was observed after 4–6 h, with no further change. At concentrations corresponding to MFCs, the cell number was reduced to zero after 2–6 h.

### 2.3. EEPs Are Effective in Eradication of Biofilm Formed by Candida Cells

In the next step, the effectiveness of EEPs in eradication of biofilm formed by *Candida* cells on abiotic surfaces (polystyrene plates, silicone and PVC (Polyvinyl Chloride) catheters) was investigated. Among the 34 clinical isolates of three species from the *Candida* genus used as model microorganisms in this experiment, the highest adherence ability was found for *C. krusei*, followed by *C. glabrata* and the lowest for *C. albicans*. As a measure of EEPs’ biofilm eradicating ability, the MBEC_50_ values, i.e., minimal concentrations causing 50% biofilm eradication, measured by the MTT assay, were determined.

**Figure 3:**
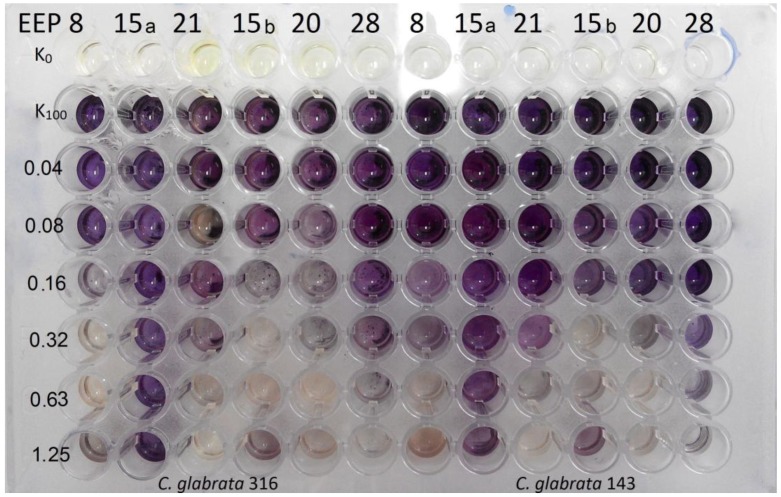
Eradication of biofilm formed by two *C. glabrata* species, visualised by the MTT assay. K_100_ indicates the growth control not affected by EEP solution, K_0_ indicates medium control (without cells and EEPs).

An example of the experiment, in which eradication of biofilm formation on polystyrene microplates by EEPs was investigated, is shown in Figure 3 and cumulative results are presented in Table 3, Table 4 and Table 5.

Generally, the biofilm eradicating activity of EEPs tested was diverse and the MBEC_50_ values were in the 0.04–1.25% (*v*/*v*) range, although the highest eradicating activity was noted for biofilm formed by *C. glabrata* and the lowest for that formed by *C. krusei*. The range of EEPs’ activity differed depending on the yeast species The range of activity assessed based on weighted average in the case of *C. albicns* was 8 > 15 b > 21 > 20 >> 28 >> 15 a, in the case of *C. glabrata* 20 > 8 ≥ 15 b > 21 > 28 >> 15 a and for *C. krusei* that was 21 > 20 > 8 > 15 b > 28 > 15 a Additionally, EEP 15 a, at the highest tested concentration (1.25% *v*/*v*) in many cases (all species), was not sufficient for biofilm eradication in 50% (Figure 3 and Table 3, Table 4 and Table 5). Controls of solvent used for propolis extraction showed that concentration of ethanol up to 10% (*v*/*v*) does not affect the ability of biofilm formation by *C. albicans* ATCC 10231 and *C. glabrata* DSM 11226 (Figure 1).

**Figure 4:**
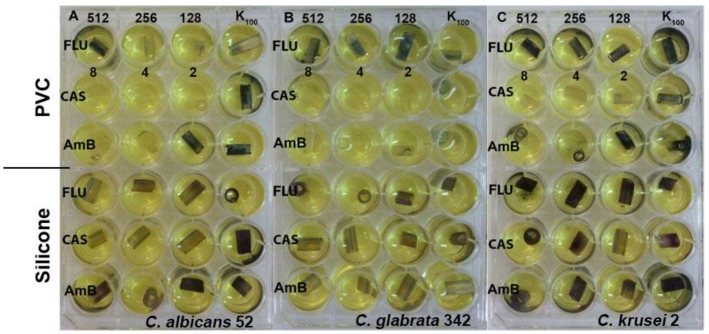
Effect of fluconazole (FLU) in the range of concentration 512–128 μg/mL, caspofungin (CAS) 8–2 μg/mL and amphotericin B (AmB) 8–2 μg/mL on eradication of biofilm formed by (**A**) *C. albicans*, (**B**) *C. glabrata* and (**C**) *C. krusei* clinical isolates from PVC and silicone catheters.

**Figure 5:**
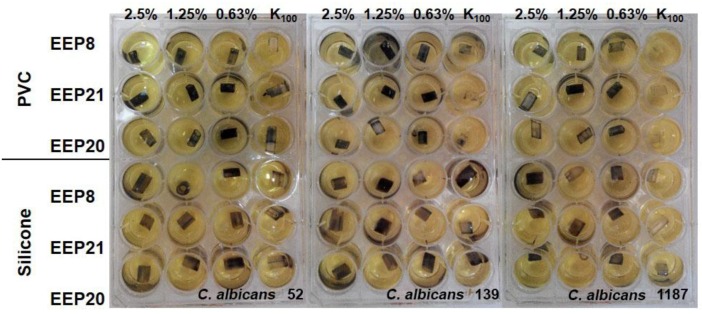
Product of MTT reaction both with living cells in biofilm and propolis extracts. K_100_ indicates the growth control not affected by EEP solution. Lack of colour in growth control indicate low ability of biofilm formation by the given isolate. Violet colour on the pieces of catheters treated with EEP within the same isolate may suggest that the coloured product results from the interaction of MTT with EEP.

**Figure 6:**
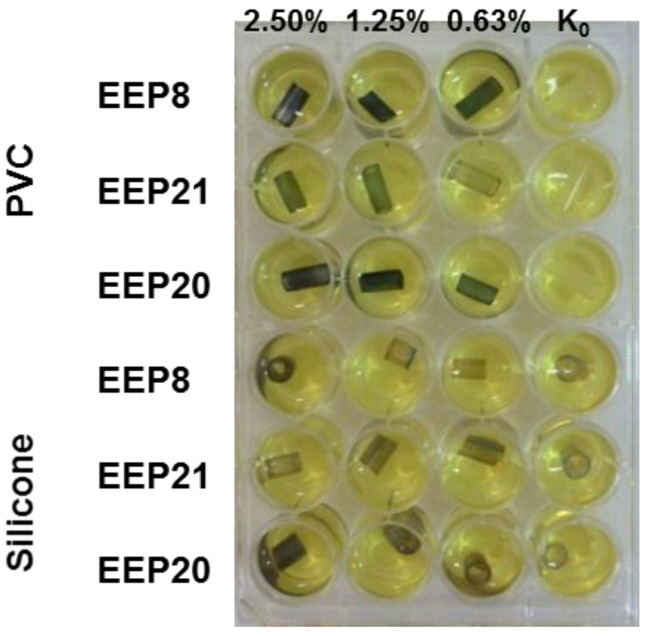
Product of interaction of MTT with propolis solution without addition of yeast cells forming biofilm structure. K_0_ indicates control pieces not treated with EEP solutions.The EEPs tested exhibited some biofilm eradicating activity, with MBEC_50_ values in the 0.63 to 5% (*v*/*v*) range; only in two cases, the MBEC_50_ value could not be determined. The most resistant biofilm was formed by *C. krusei* strains, followed by those of *C. glabrata*. In general, the biofilm eradicating potential of four EEPs tested was comparable.

Evaluation of biofilm formation by *Candida* yeasts on two types of catheters revealed that the silicone surfaces appeared to be much more prone to adhesion than the PVC ones. Using the MTT assay methodology, we investigated the biofilm eradicating activity of known antifungals, fluconazole, caspofungin and amphotericin B. Caspofungin at concentrations 8–2 μg/mL effectively eradicated fungal biofilm formed on the PVC surfaces but not those on silicone catheters. Amphotericin B was effective against *C. albicans* and *C. glabrata* biofilm formed on PVC catheters at concentrations 8–4 μg/mL. Fluconazole was ineffective at concentrations up to 512 μg/mL (Figure 4). The same methodology (MTT assay) could not be used for determination of biofilm eradicating activity of EEPs since we found that components of these extracts interact with PVC and silicone surfaces and in consequence react with MTT in the absence of yeast biofilm, thus generating false results (Figure 5 and Figure 6). Substitution of MTT with XTT allowed for eliminating the nonspecific interactions (data not shown), so that the biofilm eradication potential of EEPs was determined using the XTT-based assay. The MBEC_50_ values are shown in Table 6.

### 2.4. Combined Action of EEPs with Known Antifungals

**Figure 7:**
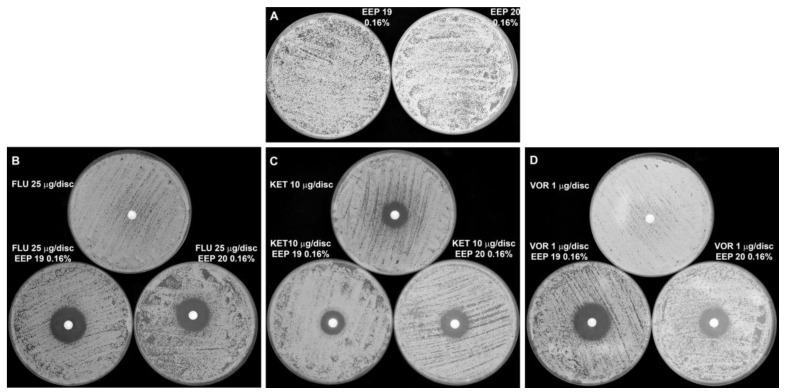
Synergistic growth inhibitory effect of EEPs in combination with azole antifungals against *C. albicans* clinical isolates. The EEPs were added to the YPD agar medium to final concentration of 0.16% (*v*/*v*). Azole antifungals were applied at paper discs. Plates were inoculated with 10^5^ cells/mL and incubated (24 h, 37 °C). (**A**)—EEPs alone. (**B**–**D**)—upper plates: azole antifungals on the discs (no EEPs in agar medium); B, C, D—lower plates: EEPs in the agar medium (EEP 19—left plate and EEP 20—right plate) and azole antifungals on the discs. Azole antifungals concentrations: fluconazole—25 µg/disc (plates B); ketoconazole—10 µg/disc (plates C); voriconazole—1 µg/disc (plates D).

In the preliminary screening, we investigated the effect of the combined action of EEPs with some known antifungals: amphotericin B (AmB), 5-fluorocytosine (5-FC), caspofungin (CAS), fluconazole (FLU), voriconazole (VOR) and ketoconazole (KET)) by the disc diffusion method. EEPs were present in agar medium at concentrations lower than their MICs and antifungals were applied on the paper discs. No interaction was noted for combinations of EEPs with AmB, 5-FC and CAS. Conversely, synergistic effects were observed for combination of EEPs with FLU or VOR. As shown in Figure 7, FLU and VOR alone did not show a growth inhibitory effect, but such effect was observed in plates containing EEPs in agar medium. Interestingly, no similar potentiation of growth inhibitory effect was observed for the KET/EEP combination (Figure 7).

Since the disc diffusion method affords only semi-quantitative results, the possible synergism between azole antifungals and EEPs was also investigated by the checkerboard method. The test microorganism in these experiments was the *C. albicans* strain resistant to FLU (MIC > 256 μg/mL supplied by The Children’s Memorial Health Institute in Warsaw). The MIC_90_ of VOR for this strain was 32 μg/mL. In the presence of EEPs, this value could be substantially reduced and the synergistic effect was observed for five out of seven EEP/VOR combinations (Table 7). Even more profound MIC reduction and stronger synergism was observed for the EEP/FLU combination (Table 8). In a second variant of the experiment, performed in 96-well microtitration plates with constant concentration of EEP and gradient of the drug in each row at the same time the reduction of efficient minimal concentration inhibiting the growth of fungi in medium containing 0.02% (*v*/*v*) of EEP 18 a was 256 and 32 times for fluconazole and voriconazole respectively (Table 9). Another, important advantage coming from the presence of EEPs in the growing medium is complete elimination of the phenomenon of residual growth of *C. albicans* at high concentration of azoles (higher than MIC or MFC).

### 2.5. Influence of EEPs on Cell Wall Formation/Integrity

To test whether EEPs may affect the fungal cell wall, MIC assays were performed in the presence of sorbitol, a known osmoprotectant. Addition of sorbitol had little effect on the MIC values of EEPs against *C. albicans*. Paradoxically, these values were even two-fold lower than those determined in medium without the osmoprotectant (Table 10). The sugar alcohol itself had no effect on fungal growth. This result suggests that EEPs do not affect the cell wall, which is an osmoprotective barrier of fungal cells. For *C. glabrata*, the effect was even more puzzling as, in the presence of both sorbitol and three of the four tested EEPs, the strain was strongly affected for growth. Different results were obtained for EEP 23 where the MIC was not affected by the addition of sorbitol (Table 10).

### 2.6. Exogenous Ergosterol Affects EEP Antifungal Action

Supplementation of the growth medium with ergosterol resulted in high (8–16×) increase of MIC values of EEPs, as shown in Table 10. This increase was comparable to that noted for the MIC of AmB, the mechanism of action of which is directly related to ergosterol binding in the cell membrane. In the case of *C. glabrata*, an increase of the MIC value was 4–16× for three out of four EEPs tested. Similarly, like in the sorbitol assay, EEP 23 behaved opposite to the other EEPs tested and its MIC in the presence of ergosterol was 4 to 8-fold lower.

### 2.7. Depolarization of Cell Membrane by EEP

**Figure 8:**
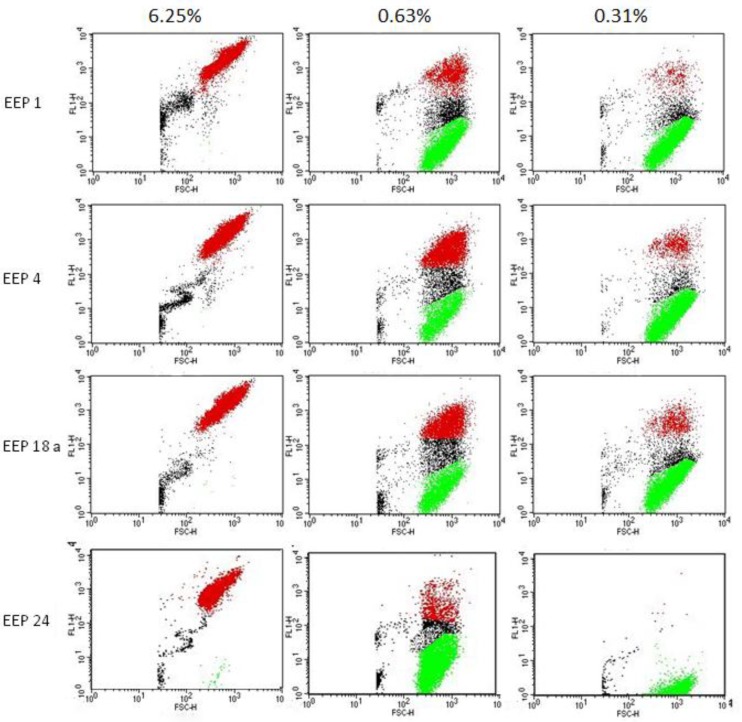
Dose dependent effect of four EEPs on *C. albicans* cell membrane depolarization. Cells were exposed to the action of EEP in the concentrations corresponding to 10× MFC, 1× MFC and ½× MFC for 1 h. Red points indicate cells with a high level of fluorescence, thus having depolarized membranes, green points with relatively low fluorescence—living cells; between these two gates, there is a group of points indicating apoptotic cells.

The aim of this experiment was to check if EEPs may cause membrane depolarization. For this purpose, anionic dye DiBAC_4_ was used. This dye is only able to enter cells with depolarized cell membrane where it binds to the intracellular proteins, which causes a significant fluorescence increase. The results of the test demonstrated that the effect of depolarization is dose dependent. At the concentration corresponding to 10× MFC, the effect of depolarization was very fast and appeared in most of the cells (up to 96% after exposition to EEP 24). At the concentration of 1× MFC, the effect was more divergent (EEP 24 caused depolarization of only 5% of cells while EEP 4 was over 40%). Some effect was also observed at a concentration of ½× MFC, especially in the case of EEP 4—about 10% (Table 11 and Figure 8). Controls of EEP solutions and medium did not increase fluorescence.

### 2.8. Effect of EEPs on Yeast-to-Mycelium Transition of C. albicans

**Figure 9:**
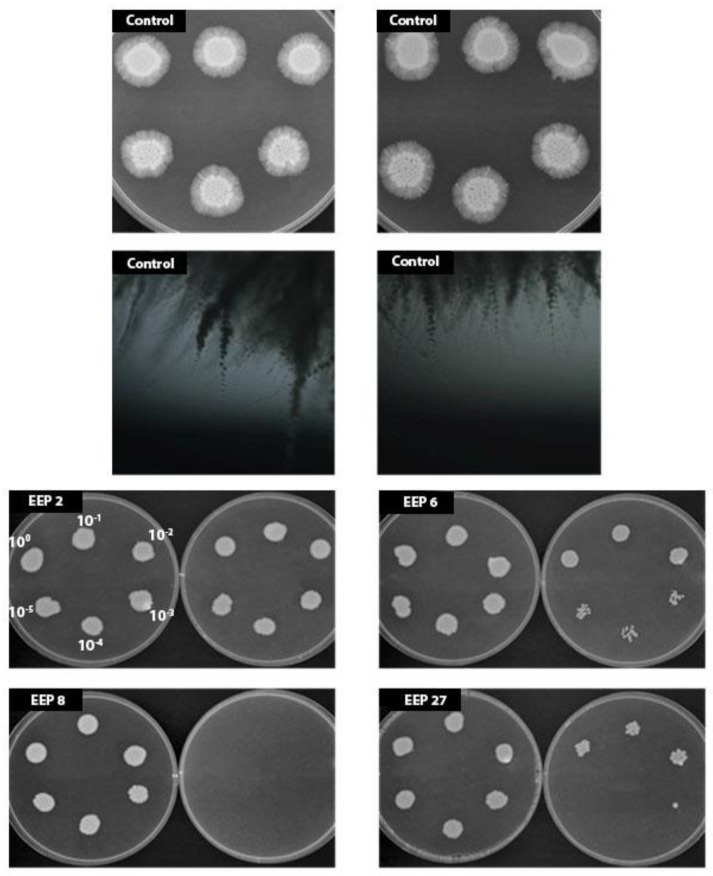
Effect of EEPs on hyphae formation by *C. albicans* SC5314 cells. Cells were grown on Spider agar without EEP (control, the bottom pictures present microscope view of the colonies) or with EEPs at concentrations 0.16% (*v*/*v*) (left dishes) or 0.31% (*v*/*v*) (right dishes) for 7 days at 37 °C. Cells were serially 10-fold diluted (10^0^–10^−5^ like int the picture EEP 2) before plating.

Hyphae formation is one of the most important virulence factors of *C. albicans* and ability to inhibit this transition is an advantageous feature of any potential antifungal drug. Effect of EEPs on yeast-to-mycelia transformation was studied in the Lee medium [11]. As shown in Table 12, after 2 h of incubation, a vast majority of cells in the control was in the form of pseudohyphae or hyphae. The percentage of hyphae forms in samples exposed to the action of EEP did not exceed 14%. Further incubation with the presence of EEP allowed reduction of mycelium forms to nearly zero. The experiment carried out in a solid Spider medium (Figure 9) revealed that addition of EPPs in concentration ½ or ¼× MIC caused inhibition of hyphae formation in all cases, in both concentrations. Therefore, undoubtedly, EEPs are effective inhibitors of *C. albicans* morphological transformation.

### 2.9. Comparison of Phenolic and Antioxidant Profiles of EEP Extracts

Three EEPs with high and one with low antifungal activity were analyzed with the use of the HPLC-DAD-MS (High-performance liquid chromatography with diode-array detection and mass spectrometry) system. The HPLC-DAD analysis revealed the presence of phenolic acids—mostly hydroxycinnamates, flavanonols, flavonones, flavonols and flavones in the extracts studied. The peaks exhibiting high UV-absorbing properties were putatively identified by LC-MS based on their mass spectra and available literature data [12]. Mass spectra of the polyphenols detected in the propolis extracts provided data about molecular weights and constitutive units of analytes detected. APCI (atmospheric chemical ionization) mass spectra of polyphenols in the positive and negative ion mode under the experimental conditions usually showed protonated molecular ions as a main peak [M + H]^+^ and [M − H]^−^, respectively (Table 13). These assignments were then confirmed by comparing HPLC-DAD retention times and UV spectra with those of authentic standards when available.

**Figure 10:**
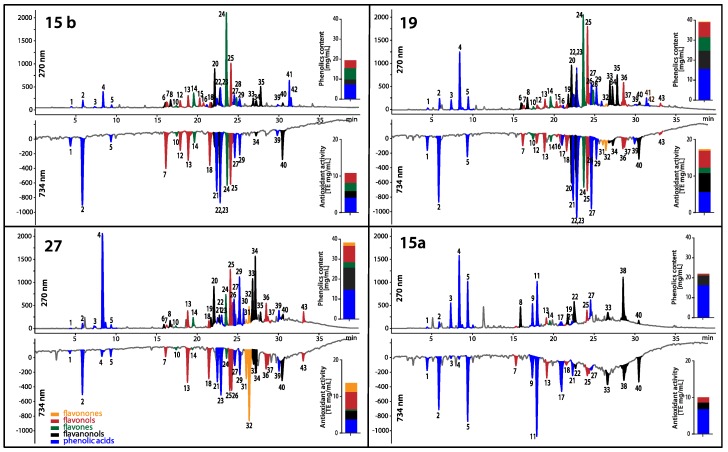
HPLC-DAD chromatograms of polyphenols (top chromatograms at 270 nm) along with profiles of antioxidants detected online with ABTS (2,2′-azino-bis(3-ethylbenzothiazoline-6-sulphonic acid) reagent (bottom chromatograms at 734 nm) in four propolis extracts (15 b, 27, 19, 15 a). The content of specific class of polyphenols and their antioxidant activity was presented as bar graphs. For identity of peaks, see Table 13. The range of antifungal activity of the EEPs was 15 b > 27 > 19 >> 15 a, and probably indicate that the presence of flavones and flavonols is crucial for antifungal activity.

The total content of phenolic compounds in EEPs studied varied from 20.4 to 41.3 mg/mL (Figure 10). The obtained chromatographic profiles of phenolics indicate differences between content of a specific class of polyphenols. The EEPs with the highest antifungal activity: 15 b, 27 and 19 had much higher content of flavones and flavonols than extract 15 a with the lowest antifungal activity. In the case of extracts 15 b, 27 and 19, the flavones and flavonols were in the range 2.6–7.1 mg/mL and 3.0–4.8 mg/mL, respectively. In addition, it can be concluded from the analysis that the activity of propolis extracts is not affected by the content of hydroxycinnamic acid derivatives, as they are contained in both active and inactive propolis (EEP 15 a) in the range from 3.7 to 6.7 mg/mL.

Chromatographic profiling coupled with chemical post-detection not only reveals the individual reducing analytes, but also enables quantitation of their input into the antioxidant potential of the sample. In Figure 10, the abundance of individual groups of polyphenols and their input into the antioxidant activity expressed as Trolox equivalents of EEP extracts studied are jointly presented. The obtaining antioxidants profiles indicate that all five detected classes of polyphenols have antioxidant activity. The total antioxidant activity of propolis samples calculated as a sum of concentration of Trolox equivalents assessed for each class of polyphenols was in the range 10.0–17.3 mg/mL. The strong correlation was observed between the total antioxidant activity and the total content of polyphenols found in the studied propolis samples, as shown by the Pearson coefficient: 0.91.

## 3. Discussion

Searching for any new ways of improvement of current antifungal therapy is the main goal of many scientific teams in the world. Natural products continue to be an inexhaustible resource of compounds of potent antimicrobial activity. In line with this tendency, we have focused our attention on ethanolic extracts of honey bee propolis (EEPs), which is known for its antimicrobial activity without serious side effects. Our previous research confirmed a high antifungal potential of propolis produced in some Polish apiaries [13]; however, a very limited number of propolis samples (*n* = 4, collected in the south part of Poland) and *Candida* spp. strains were investigated in that study. Herein, we examined activity of 50 samples of propolis obtained from different geographical origins in northern Poland against a large collection of *C. albicans* clinical isolates. The preliminary, screening tests revealed that definitely most of the investigated propolis samples exhibited promising activity against *C. albicans* and *C. glabrata* reference strains. However, some important differences in activity were observed among the samples tested. Some of them, eight in the case of *C. albicans* ATCC 10231 and three in the case of *C. glabrata* DSM 11226, were able to kill (according to MFC value) the human pathogenic yeasts of the *Candida* genus at the concentration 0.31% *v*/*v*, whilst 7 and 19 EEPs, respectively, were not able to eliminate these strains respectively at the highest tested concentration (2.5% *v*/*v*). Moreover, slightly higher resistance was observed in the case of *C. albicans* strains B4 and Gu5 (fluconazole resistant, overproducing efflux pumps) in comparison to the corresponding strains B3 and Gu4 (fluconazole sensitive, not overproducing efflux pumps). This phenomenon was not observed in our previous study and could suggest that some important active ingredients of propolis can be effectively eliminated from the yeasts’ cells overproducing drug efflux transporters. This issue still needs explanation and is a subject of our current research. The activity of six selected (the most active) EEPs was additionally tested against the group of 69 *C. albicans* clinical isolates. Most of them were killed at concentrations of 0.31 or 0.63% *v*/*v*, and resistance to the concentration of 2.5 % (*v*/*v*) was observed only in the case of one strain for only one EEP. High antifungal potential and some differences in the activity of propolis samples (due to differences of its chemical composition) collected in different regions of The World were also provided by other researchers. For example, Siqueira and coworkers stated that fungicidal activities of Brazilian red propolis were in the range of concentrations of 64–512 μg/mL for *C. albicans* and and 64–256 μg/mL for *C. glabrata* [14]. The group of Tobaldini investigated activity of Brazilian green propolis and found that MIC values for this product ranged from 220 to 880 μg/mL [15]. High antifungal potential of propolis collected in Brazil was also revealed by other authors [16,17,18]. In addition, the groups of Santos [19,20] and Pina [21] successfully treated oral candidiasis with extracts of Brazilian propolis. Haghdoost reported activity of Iran propolis for which MIC and MFC values against *C. albicans* isolates were 360.6 μg/mL and 1250.1 μg/mL, respectively [22]. The MFC values for ethanolic extracts of propolis collected in South Africa varied from 147 to 3125 μg/mL [23]. On the other hand, Massaro stated that propolis from eastern Australia was not active against *C. albicans* ATCC 10231 [24]. Taking into account the method of preparing the EEPs in our studies (extraction of dry, raw material with 70% ethanol at 3:1 (*v*/*w*), and about 20% of the raw material was removed as an insoluble waste), the mass concentration of 0.31% (*v*/*v*) solution (equal to MIC of the most active products) can be estimated as about 630 μg/mL. Thus, the activity of the propolis collected in Polish apiaries is satisfactory and comparable to the activity of the products from other geographical regions.

We also investigated the activity of our EEPs in combination with known antimycotics. Searching for a possible synergistic combination is very important because overuse of antimicrobial drugs often leads to resistance and the need for using higher and higher doses of antifungals, which can involve serious side effects. The problem of drug resistance is especially common for fluconazole, which is considered the least toxic of the currently used antifungals and thus the most extensively used. Combinational therapy with a natural compound possesses the ability to reverse fluconazole resistance and can overcome fluconazole incompetence. The synergistic activity of Brazilian propolis with fluconazole was observed by Pippi and coworkers [17]. In this study, we were able to demonstrate a synergistic effect of Polish propolis in combination with fluconazole and voriconazole. Not evident potentiation of ketoconazole activity was observed. However, this agent alone exhibited quite high activity with a relatively large zone of growth inhibition, which could cover the influence of EEP components. In addition, we proved that addition of EEP in very small concentrations, not exceeding 0.04%, can reduce the effective dose of the fluconazole up to 256 times. Another important remark, which we observed in our study, was that the addition of EEP to the medium allowed elimination of the so called ‘trailing growth’. The term ‘trailing’ has been used to describe the reduced but persistent growth that some isolates of *Candida* spp. exhibit at drug concentrations above the MIC in broth dilution tests with azole antifungal agents [25]. This phenomenon is the most prevalent in *C. albicans* isolates. According to Zomorodian et al., trailing growth appears in around 90% of isolates exposed to the activity of ketoconazole, itraconazole or fluconazole and in 78% of isolates exposed to voriconazole. Occasionally, it was also recorded for *C. tropicalis* and *C. dubliniensis* [26]. The existence of trailing growth can be the cause of recurrent infections ant treatment failure, thus the use of EEPs in combined therapy would be reasonable.

Biofilm formation by *Candida* spp. is a key factor in the survival of these cells in the host and is responsible for colonization of tissues and indwelling devices. The possible mechanisms of biofilm resistance to antimicrobial agents include impeded drug penetration through the extracellular structure, phenotypic switching and induction of the expression of resistance genes [27]. In our research, we revealed that eradication of biofilm in at least 50% requires EEPs concentrations up to 5% (*v*/*v*) (in individual cases higher). This confirms the conclusions drawn by other researchers about higher resistance of cells living in a biofilm matrix. Capoci et al. [28] tested activity of Brazilian propolis extracts toward 29 clinical isolates of *C. albicans*. They stated that concentration of EEP corresponding to ½× MIC (273.43 μg/mL) was able to reduce biofilm formation in 26% to 95%, depending on the isolate type [19]. Freires and coworkers revealed that EEPs disrupted biofilm structures of *Candida* spp. at a concentration of 500 μg/mL [18]. Thus, propolis seems to be a promising preparation in the treatment of fungal biofilm.

Another important phenomenon, phenotypic switching, is common for *C. albicans* strains, among which hyphal growth is the most common cause of invasive fungal infections in humans. Morphological plasticity is its defining feature and is critical for its pathogenesis. Unlike other fungal pathogens that exist primarily in either yeast or hyphal forms, *C. albicans* is able to switch reversibly between yeast and hyphal growth forms in response to environmental cues. Nutrient limitation is a known cue that can stimulate yeast-to-hypha morphogenesis at elevated temperatures [29]. Our results indicate that propolis extracts efficiently inhibit hyphal growth at sublethal concentration in both nutrition limitation culture media. This observation is in agreement with the results of Haghdoots and coworkers who revealed high efficiency of Iranian propolis in reduction of germ tube formation by *C. albicans* isolates [22].

Our studies provided some novel information on possible mode of action of EEPs. Reversal of this action by ergosterol and results of experiment aiming in investigation of membrane depolarization suggested that the cell membrane may be a possible site of action of EEPs components. Experiment with sorbitol as osmoprotectant agent suggests that the cell wall is not a target for ingredients of this product or is a target of secondary importance. However, the detailed mechanism of antifungal activity of EEPs remains unclear and is a subject of our current research.

## 4. Materials and Methods

### 4.1. Materials

Ethanolic extracts of 50 propolis samples (EEPs) obtained from apiaries located in northern Poland were prepared. Dry, raw material was extracted with 70% ethanol at 3:1 (*v*/*w*) for 5 days at room temperature. After this time, the extract was centrifuged (2290× *g*, 20 min) and the obtained supernatant was filtered through the sterile, 0.22 μm pore size, filters (obtained from Millipore, Burlington, MA, USA). Antifungals: fluconazole, voriconazole, ketoconazole, amphotericin B, 5-fluorocytosine, caspofungin as well as MTT, XTT, PBS, and RPMI 1640 medium were purchased from Sigma (St. Louis, MO, USA).

### 4.2. Strains

Determination of antimicrobial potential of polish EEPs was carried out using: three reference strains (*C. albicans* ATCC 10231, *C. albicans* SC5314 and *C. glabrata* DSM 11226) and 89 *Candida* spp. clinical isolates (69 strains of *C. albicans*, 10 strains of *C. glabrata* and 10 isolates of *C. krusei*). The clinical isolates were provided by two Polish hospitals: Children’s Memorial Health Institute in Warsaw and the Hospital of Medical University of Gdansk. Moreover, four *C. albicans* isolates, assigned as B3, B4, Gu4 and Gu5, were used for investigation if the components of EEPs are effectively removed from the cells by the drug efflux transporters. The strains were kindly provided by Prof. Joachim Morschhäuser from University of Würzburg (Würzburg, Germany). Strains Gu4 and B3 are fluconazole-sensitive isolates obtained from early infection episodes, while Gu5 and B4 are the corresponding fluconazole-resistant isolates obtained from later episodes in the same patients treated with fluconazole. In the case of Gu5, the lack of susceptibility to fluconazole is a consequence of overexpression of CDR1/2 genes encoding ABC transporters, whereas the resistance of B4 strain is caused by overexpression of MDR1 gene encoding a membrane transport protein of the major facilitator superfamily (MFS) [30].

### 4.3. Determination of Minimum Inhibitory Concentration (MIC) and Minimum Fungicidal Concentration (MFC)

Antifungal in vitro activity was determined by CLSI recommended microdilution method with minor modifications [31]. Briefly, serial two-fold dilutions of the tested extracts of propolis (in the range of concentrations from 0.01–5% (*v*/*v*)) were prepared in 96-well microtitration plates in the final volume of 100 μL of RPMI 1640 medium supplemented with 2% glucose and buffered to pH 7.0 with a MOPS buffer (3-*N*-morpholinopropanesulfonic acid). Suspensions of the microorganisms were prepared by taking one loop of pure culture into sterile water and adjusting optical density to 0.1 at 660 nm wave length and further 50-fold dilution in RPMI 1640 medium resulting in cell concentration of approximately 2 × 10^4^ CFU/mL. 100 μL of such suspension was inoculated to each well of the microtitration plate leaving agent-free column as a growth control and agent and cell-free column as sterility controls. As a consequence of inoculation, the final range of EEPs’ concentration was from 0.005 to 2.5% (*v*/*v*). The plates were incubated for 24 h at 37 °C. In some cases, EEPs after contact with medium created sediment (especially in the highest concentrations) that could be misinterpreted as cell growth, so, in this paper, we present the results of minimum fungicidal concentrations (MFC) for the evaluation of the activity of gathered samples toward reference strains as well as selected EEPs towards the whole group of clinical isolates. Nevertheless, in experiments concerning modes of action, the MFC concentrations would prevent the growth of microorganisms, thus we used concentration corresponding to MIC values, which are usually twice as low as MFC. For determination of minimum fungicidal concentrations (MFC), small aliquots of suspensions (around 5 μL) from each well were transferred using the 48-well metal pinner to YPD agar plates without inhibitors and incubated for 24 h at 37 °C. Minimal concentrations of EEPs at which no growth of the colonies was observed were assigned as MFCs.

### 4.4. Eradication of Biofilm Formed on Polystyrene Plates

For checking activity of EEPs towards cells living in biofilm, we used the MTT assay and determined the MBEC_50_ value (Minimal Biofilm Eradication Concentration that causes 50% reduction of the activity of the enzyme that converts MTT to violet formazan). From overnight cultures in Sabouraud liquid medium, cell suspensions at optical densities OD_660_ = 0.1 (around 10^6^ CFU/mL) were prepared and diluted twice in sterile water. In addition, 96-well tissue culture microtitration plates containing 100 μL of 2× concentrated RPMI 1640 medium were inoculated with 100 μL of cell culture. Biofilm structure was formed for 24 h. Subsequently, wells were washed three times with PBS solution and filled with 200 μL of EEP solutions in RPMI 1640 at concentrations ranging from 0.04 to 1.25% (*v*/*v*). One column of each plate was left as a control. After 24 h incubation at 37 °C, wells were washed twice with PBS. Next the wells were filled with 90 μL of MTT solution (5 mg/mL in PBS) and left for 2 h at 37 °C. Next, the solution was removed and formed formazan was dissolved in 100 μL of isopropanol alcohol. Intensity of solubilized formazan colour was measured using a Victor3 Plate reader (Perkin Elmer, Waltham, MA, USA) at the wavelength 490 nm.

### 4.5. Eradication of Biofilm Formed on Catheters

The assays were carried out on two types of medical catheters based on polyvinylchloride or silicone (both purchased from Zarys, Zabrze, Poland). The activity of EEP toward biofilm formed on the surfaces of catheters was evaluated against a group of 9 clinical isolates (3 *C. albicans*, 3 *C. glabrata*, 3 *C. krusei*) and compared to the activity of fluconazole, caspofungin and amphotericin B. EEPs concentration range was 2.5–0.63% (*v*/*v*), fluconazole 512–128 μg/mL, amphotericin B and caspofungin 8–2 μg/mL. Fragments of catheters were cut into 0.5 cm pieces and placed in 24-well plates. Each well was filled with 1 mL of 2× concentrated RPMI 1640 medium and 1 mL of cell suspension (5 × 10^5^ CFU/mL of water). Biofilm structure was formed for 24 h. Fragments of catheters were washed with PBS and placed in 2 mL of fresh solutions of EEPs or antifungals in RPMI 1640 medium. After 24 h of incubations catheters were again rinsed with PBS and transferred to new 24-well plates containing either 2 mL of MTT (1 mg/mL in Ringer solution) or XTT (0.5 mg/mL in Ringer solution with the addition of 1 μL of menadione (10 mM in acetone) per 10 mL of the XTT solution just before carrying out the assay). Biomaterial fragments were incubated with MTT or XTT solutions for 24 h followed by their visual observations. Additionally, XTT solution intensity colour was measured with Victor3 Plate reader (Perkin Elmer, Waltham, MA, USA) at the wavelength 450 nm. The MBEC_50_ values were determined.

### 4.6. Time-Kill Assay

Cells from the overnight culture (16–18 h) on YPD agar plates were suspended in sterile water and OD_660_ was adjusted to about 0.1 (around 10^6^ cells per 1 mL). The suspension was then diluted 10-fold with RPMI 1640 medium and EEPs were added at concentrations corresponding to ½× MFC or MFC. Control (without addition of EEPs) and treated samples were incubated at 37 °C for 0.5, 2, 4, 6 and 24 h with shaking. To avoid the carry over effect, after the appropriate time of incubation, 1 mL of each suspension was centrifuged (2 min, 9170× *g*) and the pellet was re-suspended in 1 mL of PBS pH 7.4. Ten-fold serial dilutions with PBS were prepared and 100 μL of each was inoculated on YPD agar plates. Plates were incubated for 24 h at 37 °C. Colony forming units in the range 30–300 were counted and the number of cells in 1 mL (CFU/mL) was calculated.

### 4.7. Determination of Effects of Combined Action of EEPs with Common Antifungal Drugs

Preliminary test by disc diffusion method. Suspensions of 10^5^ *C. albicans* cells/mL were spread on the surface of YPD agar plates containing 0.16% (*v*/*v*) of EEP (plates without EEPs were used as controls). Paper discs (5 mm diameter; Oxoid) saturated with solutions of antifungal drugs (amphotericin B (AmB), 5-fluorocytosine (5-FC), caspofungin (CS), fluconazole (FLU), voriconazole (VOR) and ketoconazole (KET)) were placed centrally on the agar surface. Plates were incubated for 24 h at 37 °C and diameters of growth inhibition zones were measured.

Determination of fractional inhibitory concentrations by checkerboard method. After overnight culture (16–18 h) on YPD agar plates, colonies of the *C. albicans* fluconazole resistant clinical isolate (one of the strains supplied by the Children’s Memorial Health Institute in Warsaw) were suspended in sterile water. Cell number in the suspension was adjusted to 10^6^ cells per 1 mL and the inoculum was diluted 50-fold to obtain the cell density of 2 × 10^4^ CFU/mL. A gradient of antifungal chemotherapeutic was established along the horizontal axis and EEP (no 18 b or 23) along the vertical axis. The first row contained only the gradient of chemotherapeutics in the range of concentration 256–1.0 μg/mL for fluconazole and 32–0.125 μg/mL for voriconazole. The tenth column contained a gradient of the EEP in the range 0.000625% to 0.08% (*v*/*v*), while columns 1–9 were in the range 0.00125% to 0.08%. In addition, 100 μL of the prepared suspension was inoculated to each well of the plate. Plates were incubated for 24 h at 37 °C. MIC values of the compound alone or in combination were read with the Victor3 Plate Reader (Perkin Elmer, Waltham, MA, USA). ƩFIC (fractional inhibitory concentration) was determined according to the equation ΣFIC = FIC A + FIC B, where FIC A is the MIC of the drug in combination/MIC of the drug alone, FIC B is the MIC of the EEP in combination/MIC of the EEP alone. The combination is considered synergistic when the ΣFIC is ≤0.5, indifferent when the ΣFIC value is between 0.5 and 4.0, and antagonistic when the ΣFIC is ≥4.0 [32].

In the second method, the MIC values of fluconazole and voriconazole were determined at three different concentrations of EEPs (0.01, 0.02, 0.04% (*v*/*v*)). The test was performed in 96-well microtitration plates. The gradient of fluconazole or voriconazole was established along the horizontal axis in the range 512–1 μg/mL and 8–0.015 μg/mL, respectively. For both drugs, the gradient was prepared in four successive rows. Next, the medium in the rows was supplemented with EEPs. The first row was used as a control and contained only the gradient of drug alone, the second row contained the addition of 0.01% of EEP, the third 0.02% of EEP and the fourth 0.04% (*v*/*v*) of EEP. The plates were incubated for 24 h at 37 °C and results were read visually. The MIC was established as the concentration where no growth was observed.

### 4.8. Determination of Sorbitol Effect on Anti Candidal Activity of EEPs

The MIC values of EEPs were determined in RPMI-1640 growth medium containing 8.0 M sorbitol as an osmoprotectant. Gradient of EEPs was prepared with two-fold broth microdilution method and plates were inoculated with cell suspension to the final cell density of 10^4^ CFU/mL. Plates were incubated 24–48 h at 37 °C. All traits were also investigated for MFC values, according to the method described in Section 2.1.

### 4.9. Determination of Ergosterol Effect on Anti Candidal Activity of EEPs

MIC values were determined according to CLSI guidelines [29] in medium supplemented with ergosterol. Medium with ergosterol was prepared at the time of the test. The test was performed in microtitration plates as in the case of the routine MIC determination procedure. Ergosterol powder was dissolved in DMSO (dimethyl sulfoxide) containing Tween 80 (1% *v*/*v*) to the final concentration of no more than 10% (*v*/*v*). The obtained solution was added to RPMI-1640 medium through the 0.2 μm filter, to the final ergosterol concentration of 100 μg/mL. Two-fold serial dilutions of EEPs (concentration in the range 5–0.01% (*v*/*v*) were prepared in the RPMI-1640 medium supplemented or not supplemented with ergosterol. Plates were inoculated with cell suspension to the final cell density of 10^4^ CFU/mL and incubated for 24–48 h at 37 °C. Amphotericin B was used instead of EEPs, as a positive control. Investigations were carried out in duplicate. The MIC values were read as the lowest concentration of EEPs or amphotericin B where no growth of yeast was observed. Additionally, the MFC values were determined by picking up small amounts of suspension from each well and culturing on EEP-free solid YPD medium.

### 4.10. Depolarization of Cell Membrane by EEP

*C. albicans* SC5314 strain was refreshed on YPD agar medium (24 h, 30 °C). Few colonies were transferred to LowFlow medium and incubated overnight (30 °C, 240 RPM). Cell density was adjusted to 0.5–1.0 (OD_600_) and resulted suspension was incubated for a further 3 h (30 °C, 240 RPM). Solutions of EEPs in the concentrations 6.25%, 0.63% and 0.31% (*v*/*v*) corresponding to 10× MFC, 1× MFC and ½× MFC were added to the samples. At the same time, controls of EEPs’ solutions, LowFlow medium and cell suspension were prepared. Incubation was continued for one hour more. Suspensions were centrifuged (825× *g*, 5 min), washed with sterile water and OD_600_ = 3.0 was established. Samples were diluted approximately 50 times and stained for 15 min with DiBAC_4_ (Bis-(1,3-Dibutylbarbituric Acid) Trimethine Oxonol) dye in dark (final concentration 1.6 μg/mL). Fluorescence rate was measured with a FACScan cytometer (Becton Dickinson, Franklin Lakes, NJ, USA). Excitation length was 490 nm and emission 530 (±15 nm).

### 4.11. Yeast to Mycelia Morphological Transition

*C. albicans* SC5314 cells were grown overnight in Sabouraud liquid medium (150 RPM, 30 °C). Cells were washed twice with sterile water and suspended in Lee medium [11] to cell density 10^6^ cells per mL. EEPs were added to the final concentration corresponding to ½ MIC or MIC and cell suspensions were cultivated at 37 °C. Samples of cell suspensions were withdrawn at appropriate time intervals and a percentage of mycelial forms was estimated in a Thoma cell counting chamber. Additionally, inhibition of hyphae formation was evaluated on Spider agar. Medium consisted of nutrient broth 1% (*w*/*v*), mannitol 1% (*w*/*v*), K_2_HPO_4_ 0.2% (*w*/*v*) and agar 1.35% (*w*/*v*). EEPs were added at concentrations around ½× MIC or ¼× MIC. Samples of cell suspension (5 μL, 10^6^ cells per mL and 5 further 10-fold dilutions) were spotted on Spider agar plates and the micromorphology of growing colonies (mycelium formation) was monitored daily for 7 days of incubation at 37 °C. The presence of mycelial growth at the colony edges was determined using a light microscope and photographed using a digital camera.

### 4.12. Phenolic Content and Online Antioxidant Activity Determination

The HPLC system applied for phenols determination consisted of an Agilent 1200 series photodiode array detector and mass spectrometer (Agilent 6130 Quadrupole LC/MS, Santa Clara, CA, USA) equipped with an electrospray ionization interface. A Phenomenex Kinetex XB-C18 100A column (150 × 4.6 mm) (Torrance, CA, USA) with 5 μm particle size and 4 μL injection volumes were used. The mobile phase contained aqueous 4.8% *v*/*v* formic acid (A) and methanol (B). The gradient applied was 20–100% B in 35 min and stay at 100% B for 5 min at a flow rate of 0.8 mL/min. Absorbance spectra were recorded between 190 and 400 nm every 2 s with a bandwidth of 4 nm, while the chromatograms were monitored at 270, 300 and 325 nm for phenolic acids, at 290 nm for flavanonols and flavonones, at 350 nm for flavonols and at 335 nm for flavones. MS parameters were as follows: capillary voltage, 3000 V; fragmentor, 120 V; drying gas temperature, 350 °C; gas flow (N_2_), 12 L/min; nebulizer pressure, 35 psig. The instrument was operated in positive and negative ion mode, scanning from *m*/*z* 100 to 1000. The peaks were identified by comparison of retention times and UV spectra with those of authentic reference substances or on the basis of available literature data and mass spectra. The quantification of the analytes for which standards were available was performed with external calibration curves generated by integration of the areas of absorption peaks, whereas for analytes for which standards were lacking by reporting the measured chromatographic area in the calibration equation of the reference standards (hydroxycinnamates were quantified as caffeic acid at 300 nm, flavanonols as taxifolin at 290 nm; flavonols as kaempferol at 350 nm, flavones as luteolin at 335 nm and flavonones as naringenin at 290 nm).

The profiling of antioxidants in propolis extracts was performed by postcolumn derivatization as described earlier [33,34,35] with slight modifications. The postcolumn addition of ABTS derivatization reagent to HPLC eluate was performed using a Pinnacle PCX derivatization instrument (Pickering Laboratories. Inc., Mountain View, CA, USA). The derivatization reagent was prepared by dilution of ABTS stock solution (7 mmol/L) with methanol to a concentration of 30% (*v*/*v*). Derivatization was carried out at 130 °C with the flow rate of derivatization reagents set at 0.2 mL/min. The 0.5 mL (PTFE, 0.25 mm, 10 m) coil available as a standard part of the Pinnacle PCX derivatization instrument was used. Chromatograms of the products formed after derivatization of antioxidant compounds with ABTS reagent were registered at 734 nm using a multiple-wavelength detector (Agilent 1200 series MWD, San Diego, CA, USA). The antioxidant activity of identified group of phytochemicals was assessed as a sum of concentrations of Trolox equivalent (TE) calculated with the use of equation of Trolox calibration curve and area under the negative peaks of analytes recorded in HPLC analyses during post-column derivatisation with ABTS reagent.

## 5. Conclusions

In conclusion, our research revealed that propolis produced in apiaries located in the northern part of Poland exhibits considerable antifungal activity. The ethanolic extract of this natural product effectively eliminates biofilm as well as planktonic cells of *Candida* spp. The observed synergistic effect of EEPs with fluconazole and voriconazole is especially interesting from the clinical point of view. Moreover, EEPs inhibit the formation of hyphal forms of *C. albicans*, an important virulence factor of these pathogenic yeasts. Some evidence of our investigations suggest the fungal cell membrane as the most probable target of EEP ingredients.

## Figures and Tables

**Table 1:** Activity of investigated EEPs against reference strains of *Candida* spp.

MFC (% *v*/*v*)	Reference Strains Tested						Total
	*C. albicans* ATCC 10231	*C. glabrata* DSM 11226	B3	B4	Gu4	Gu5	
>2.50	7	19	9	15	5	15	70
2.50	8	5	10	4	10	5	42
1.25	12	14	9	7	10	8	60
0.63	15	9	17	22	19	21	103
0.31	8	3	5	2	6	1	25
Number of EEPs tested	50	50	50	50	50	50	300

EEP—Ethanolic Extract of Propolis, MFC—Minimum Fungicidal Concentration, ATCC—American Type Culture Collection, DSM—Deutsche Sammlung von Mikroorganismen.

**Table 2:** Activity of six selected most active EEPs against 69 *C. albicans* clinical isolates.

MFC (% *v*/*v*)	17	21	27	15 b	18 b	23
<0.08	0	0	0	1	0	0
0.08	0	1	0	1	1	0
0.16	0	3	1	17	7	4
0.31	16	18	20	28	23	19
0.63	34	38	34	20	33	34
1.25	17	9	14	1	5	12
2.5	2	0	0	0	0	0
>2.5	0	0	0	1	0	0

**Table 3:** MBEC_50_ for 14 clinical isolates of *C. albicans*.

MBEC_50_ (% *v*/*v*)	EEPs No					
	8	15 a	21	15 b	20	28
0.04	5	3	3	2	3	2
0.08	0	0	0	1	1	1
0.16	0	0	1	2	2	0
0.31	4	1	2	5	2	0
0.63	3	1	4	1	1	3
1.25	1	0	3	1	2	2
>1.25	1	9	1	2	3	6

**Table 4:** MBEC_50_ for 10 clinical isolates of *C. glabrata*.

MBEC_50_ (% *v/v*)	EEPs No					
	8	15 a	21	15 b	20	28
0.04	2	0	0	0	2	0
0.08	1	1	3	5	4	0
0.16	6	0	2	2	3	2
0.31	0	3	3	2	1	5
0.63	1	1	1	1	0	2
1.25	0	2	1	0	0	0
>1.25	0	3	0	0	0	1

**Table 5:** MBEC_50_ for 10 clinical isolates of *C. krusei*.

MBEC_50_ (% *v*/*v*)	EEPs No					
	8	15 a	21	15 b	20	28
0.04	0	0	0	0	1	1
0.08	2	1	1	3	1	0
0.16	4	1	2	1	5	0
0.31	1	1	3	0	2	2
0.63	2	0	4	2	0	2
1.25	0	0	0	0	0	1
>1.25	1	7	0	4	1	4

**Table 6:** Eradication of biofilm formed by three *Candida* species on the surfaces of PVC and silicone medical catheters. Results obtained with XTT assay.

Clinical Isolate	MBEC_50_ (% *v*/*v*)							
	PVC				Silicone			
	EEP 2	EEP 6	EEP 8	EEP 27	EEP 2	EEP 6	EEP 8	EEP 27
*C. albicans* 52	5	> 5	5	5	0.63	0.625	0.63	0.63
*C. albicans* 139	*	*	*	*	*	*	*	*
*C. albicans* 1187	*	*	*	*	5	5	2.5	2.5
*C. glabrata* 137	1.25	0.63	1.25	1.25	0.63	0.63	0.63	2.5
*C. glabrata* 143	5	5	2.5	5	5	2.5	2.5	2.5
*C. glabrata* 342	0.63	1.25	0.63	0.63	0.63	>5	0.63	2.5
*C. krusei* 2	5	5	5	1.25	2.5	2.5	2.5	1.25
*C. krusei* 35	2.5	5	5	5	5	1.25	1.25	1.25
*C. krusei* 59	2.5	5	5	5	5	2.5	2.5	1.25

* Weak biofilm formation, XTT—(2,3-Bis-(2-Methoxy-4-Nitro-5-Sulfophenyl)-2H-Tetrazolium-5-Carboxanilide).

**Table 7:** Dose-dependent interaction of voriconazole (VOR) and EEP against *C. albicans* clinical isolate resistant to fluconazole. Results obtained by the checkerboard method.

MIC Alone		MIC in Combination		FIC of VOR in Combination	FIC of EEP in Combination	∑ FIC	Interaction ^a^
VOR (μg/mL)	EEP 18 a (% *v*/*v*)	VOR (μg/mL)	EEP 18 a [% *v*/*v*]				
32	0.08	8	0.00125	0.25	0.02	0.27	S
		8	0.0025	0.25	0.03	0.28	S
		4	0.005	0.13	0.06	0.19	S
		4	0.01	0.13	0.13	0.25	S
		4	0.02	0.13	0.25	0.38	S
		0.125	0.04	0.00	0.50	0.50	I
		0.125	0.08	0.00	1.00	1.00	I

^a^ I—indifferent, S—synergistic, FIC—Fractional Inhibitory Concentration.

**Table 8:** Dose-dependent interaction of fluconazole (FLU) and EEP against the *C. albicans* clinical isolate resistant to fluconazole. Results obtained by the checkerboard method.

MIC Alone		MIC in Combination		FIC of FLU in Combination	FIC of EEP in Combination	∑ FIC	Interaction ^a^
FLU (μg/mL)	EEP 18 a	FLU [μg/mL]	EEP 18 a (% (*v/v*))				
>256	0.08	256	0.00125	0.50	0.02	0.52	I
		128	0.0025	0.25	0.03	0.28	S
		64	0.005	0.13	0.06	0.19	S
		32	0.01	0.06	0.13	0.19	S
		32	0.02	0.06	0.25	0.31	S
		32	0.04	0.06	0.50	0.56	I
		1	0.08	0.00	1.00	1.00	I

^a^ I—indifferent, S—synergistic.

**Table 9:** Synergism of EEP with fluconazole (FLU) or voriconazole (VOR). The test was performed in 96-well microtitration plates against the *C. albicans* clinical isolate resistant to fluconazole.

EEP 18 a (% (*v/v*))	MIC_90_ (μg/mL)	
	FLU	VOR
0	>512	>8
0.01	128	16
0.02	4	0.5
0.04	4	0.5

**Table 10:** Influence of ergosterol or sorbitol on anti *Candidal* in vitro activity of EEPs.

	MIC (μg/mL)					
	*C. albicans* ATCC 10231			*C. glabrata* DSM 11226		
EEP	RPMI	RPMI + Ergosterol	RPMI + Sorbitol	RPMI	RPMI + Ergosterol	RPMI + Sorbitol
15 b	0.16	2.5	0.08	0.04	0.16	n/0.01 ^1^
17	0.31	5	0.16	0.63	>5	0.04
18 b	0.31	2.5	0.16	0.63	5	n/0.01
23	0.31	5	0.16	0.31	0.04/0.08	0.04/0.31
AmB	0.06	0.5	-	0.06	8	-

^1^ n/0.01—no entire growth (only residual growth at the given concentration of EEP).

**Table 11:** Relative fluorescence level of *C. albicans* SC5314 cells after exposition to EEP and dyeing with DiBAC_4_.

Sample	6.25%	0.63%	0.31%	Control
EEP 1	86.20	19.21	3.84	0.22
EEP 4	86.54	41.13	9.3	
EEP 18 a	90.39	27.04	7.62	
EEP 24	96.92	5.93	0.07	

**Table 12:** Number of cells forming hyphae or pseudohyphae forms after 2 or 24 h incubation with selected EEP. *C. albicans* SC5314 cells were grown in Lee medium and counted in cell counting Thoma chamber.

Sample	Hyphae and Pseudohyphae Forms (%)	
	After 2 h	After 24 h
Control	91	66
EEP 4	3	1
EEP 8	14	2
EEP 17	9	2
EEP 18 b	13	0

**Table 13:** Composition of polyphenols detected in EEPs (no 15 a, 15 b, 19, 27) with spectroscopic and spectrometric characteristics.

Peak No	t_R_ (min)	Class of Phenolics	λ_max_ (nm)	(M + H)^+^ *m*/*z*	(M − H)^−^ *m*/*z*	MW	Tentative Identification
1	4.5	PA	280, 310	139	137	138	Hydroxybenzoic acid
2	6.3	PA	292, 322	181	179	180	Caffeic acid
3	7.6	PA	280, 310	153	198	152	Metoxybenzoic acid
4	8.7	PA	310	165	163	164	*p*-Coumaric acid
5	9.7	PA	295 sh, 322	195	193	194	Ferrulic acid
6	16.4	PA	285	287	285	286	Pinobanksin-5-methyl ether
7	16.7	PA	256, 370	303	301	302	Quercetin
8	17.1	PA	292	273	271	272	Pinobanksin
9	17.8	PA	262	133	399		Unknown
10	17.9	FON	253, 268 sh, 349	287	285	286	Luteolin
11	18.3	PA	265, 300 sh	163	357		Unknown
12	18.3	FOL	256, 355	317	315	316	Quercetin-3-methyl ether
13	19.3	FOL	265, 364	287	285	286	Kaempferol
14	20.0	FON	268, 337	271	269	270	Apigenin
15	20.8	FOL	265, 352	301	299	300	Kaempferol-methyl ether
16	21.2	FOL	265, 340	331	329	330	Kaempferol-methoxy-methyl ether
17	21.4	PA	295 sh, 323	465	441	442	Ferrulic acid derivative
18	22.1	FOL	256, 367		315	316	Rhamnetin
19	22.2	FNOL	288	287	285	286	Pinobanksin-5-methyl ether
20	22.6	FNOL	289	257	255	256	Pinocembrin
21	22.7	PA	298, 325	249	247	248	Caffeic acid isoprenyl ester
22	23.1	FNOL	292	315	313	314	Pinobanksin-3-*O*-acetate
23	23.1	PA	295, 325	285	283	284	Caffeic acid phenylethyl ester
24	23.9	FON	268, 331		283	284	Acacetin
25	24.6	FOL	265, 300 sh, 358	271	269	270	Galangin
26	24.7	FOL	265, 364	301	299	300	Kaempferide
27	24.9	PA	315	251	249	250	Unknown
28	25.2	FON	265, 300 sh, 350 sh	285	283	284	6-Methoxychrysin
29	25.6	PA	295, 324		295	294	Caffeic acid cinnamyl ester
30	25.8	PA	295, 324	297	295	296	Caffeic acid cinnamyl ester
31	26.1	FONN	295	271	269	270	3-Hydroxy-5-methoxyflavanone
32	26.5	FONN	288	273	271	272	Naringenin
33	26.9	FNOL	292, 330 sh	329	327	328	Pinobanksin-3-*O*-propionate
34	27.2	FNOL	292	343	341	340	Pinobanksin-3-*O*-butyrate
35	27.8	FNOL	270, 310 sh	357	355	356	Pinobanksin-3-*O*-penatnoate
36	28.6	FOL	267, 305, 355				Unknown
37	28.8	FOL	267, 305, 355				Unknown
38	28.8	FNOL	280				Unknown
39	30.2	PA	292, 322		399	400	Caffeic acid derivative
40	30.6	FNOL	270, 290	393	369	370	Pinobanksin-5-methyl ether-3-*O*-pentanoate
41	31.1	PA	280	295	293	294	Methoxy-cinnamic acid cinnamyl ester
42	31.6	PA	280	295	293	294	Methoxy-cinnamic acid cinnamyl ester
43	33.2	FOL	267, 305, 355				Unknown

PA, Phenolic acids; FON, Flavones; FOL, Flavonols; FNOL, Flavanonols; FONN, Flavonones.
